# PPAR-α Deletion Attenuates Cisplatin Nephrotoxicity by Modulating Renal Organic Transporters MATE-1 and OCT-2

**DOI:** 10.3390/ijms21197416

**Published:** 2020-10-08

**Authors:** Leandro Ceotto Freitas-Lima, Alexandre Budu, Adriano Cleis Arruda, Mauro Sérgio Perilhão, Jonatan Barrera-Chimal, Ronaldo Carvalho Araujo, Gabriel Rufino Estrela

**Affiliations:** 1 Departamento de Biofísica, Universidade Federal de São Paulo, São Paulo 04039032, Brazil; lcf.lima@gmail.com (L.C.F.-L.); alexandre.budu@unifesp.br (A.B.); arruda_adriano@hotmail.com (A.C.A.); maurospersonal3@gmail.com (M.S.P.); araujo.ronaldo@unifesp.br (R.C.A.); 2 Departamento de Medicina, Disciplina de Nefrologia, Universidade Federal de São Paulo, São Paulo 04039032, Brazil; 3 Instituto de Investigaciones Biomédicas, Universidad Nacional Autónoma de México, Mexico City 04510, Mexico; jbarrera@biomedicas.unam.mx; 4 Unidad de Investigación UNAM-INC, Instituto Nacional de Cardiología Ignacio Chávez, Mexico City 14080, Mexico; 5 Departamento de Oncologia Clínica e Experimental, Disciplina de Hematologia e Hematoterapia, Universidade Federal de São Paulo, São Paulo 04037002, Brazil

**Keywords:** cisplatin nephrotoxicity, PPAR-alpha, organic transporters

## Abstract

Cisplatin is a chemotherapy drug widely used in the treatment of solid tumors. However, nephrotoxicity has been reported in about one-third of patients undergoing cisplatin therapy. Proximal tubules are the main target of cisplatin toxicity and cellular uptake; elimination of this drug can modulate renal damage. Organic transporters play an important role in the transport of cisplatin into the kidney and organic cations transporter 2 (OCT-2) has been shown to be one of the most important transporters to play this role. On the other hand, multidrug and toxin extrusion 1 (MATE-1) transporter is the main protein that mediates the extrusion of cisplatin into the urine. Cisplatin nephrotoxicity has been shown to be enhanced by increased OCT-2 and/or reduced MATE-1 activity. Peroxisome proliferator-activated receptor alpha (PPAR-α) is the transcription factor which controls lipid metabolism and glucose homeostasis; it is highly expressed in the kidneys and interacts with both MATE-1 and OCT-2. Considering the above, we treated wild-type and PPAR-α knockout mice with cisplatin in order to evaluate the severity of nephrotoxicity. Cisplatin induced renal dysfunction, renal inflammation, apoptosis and tubular injury in wild-type mice, whereas PPAR-α deletion protected against these alterations. Moreover, we observed that cisplatin induced down-regulation of organic transporters MATE-1 and OCT-2 and that PPAR-α deletion restored the expression of these transporters. In addition, PPAR-α knockout mice at basal state showed increased MATE-1 expression and reduced OCT-2 levels. Here, we show for the first time that PPAR-α deletion protects against cisplatin nephrotoxicity and that this protection is via modulation of the organic transporters MATE-1 and OCT-2.

## 1. Introduction

Cisplatin is a very effective drug against solid tumors. However, severe side effects have been reported [1,2]. Cisplatin-induced nephrotoxicity, which is usually dose-dependent, affects about one-third of patients undergoing cisplatin treatment [1,2]. Some animal studies have shown that cisplatin accumulates in the kidney more than in other organs [3,4,5]. It affects the proximal tubules of the kidneys by different mechanisms, such as oxidative stress, inflammation, DNA damage and apoptosis [1,2]. The initial step for cisplatin nephrotoxicity is entering the cells; some authors have suggested that the cellular uptake of cisplatin is mediated, in part, by transport proteins [6,7]. Thus, organic cation transporters (OCTs) play a role in cisplatin transport into the kidneys [8]. OCTs are located at basolateral membranes and are highly expressed in the kidneys [8,9]. Cisplatin interacts preferably with OCT-2 [10], and the inhibition or deletion of OCT-2 attenuates cisplatin nephrotoxicity [11,12,13]. Multidrug and toxin extrusion 1 (MATE-1) transporter is a protein involved in cisplatin secretion into the urine, which is localized at apical membrane [14,15]. MATE-1 deletion in mice exacerbates cisplatin nephrotoxicity [16], whereas increased MATE-1 expression decreases platinum accumulation in renal cells after cisplatin treatment [17]. Peroxisome proliferator-activated receptor alpha (PPAR-α) is a transcription factor that controls fatty acid oxidation and glucose homeostasis, and it is highly expressed in the liver and kidneys [18,19]. Our group recently showed that PPAR-α interacts with both *MATE-1* and *OCT-2* [20]. Considering that the modulation of organic transporters is an important mechanism to either increase or attenuate cisplatin nephrotoxicity, we investigated the effect of PPAR-α deletion on cisplatin nephrotoxicity severity and whether these effects are mediated by modulation of organic transporters.

## 2. Results

### 2.1. PPAR-α Deletion Attenuates Cisplatin-Induced Renal Injury

We treated C57BL6 and PPAR-α-deficient mice with a single dose of cisplatin (20 mg/kp i.p). Ninety-six hours after cisplatin (CP) treatment, the wild-type mice showed increased serum creatinine and urea levels, while PPAR-α knockout mice (CP PPARKO) avoided the increase of these parameters (Table 1. Moreover, real-time PCR was performed in the kidney to assess renal injury markers. Cisplatin treatment (CP) upregulated the mRNA levels of NGAL and KIM-1 and PPAR-α ablation (CP PPARKO) attenuated the upregulation of these molecules (Table 1).

### 2.2. PPAR-α Deletion Blunts Renal Expression of Inflammatory and Apoptosis-Related Genes

**Figure 1:**
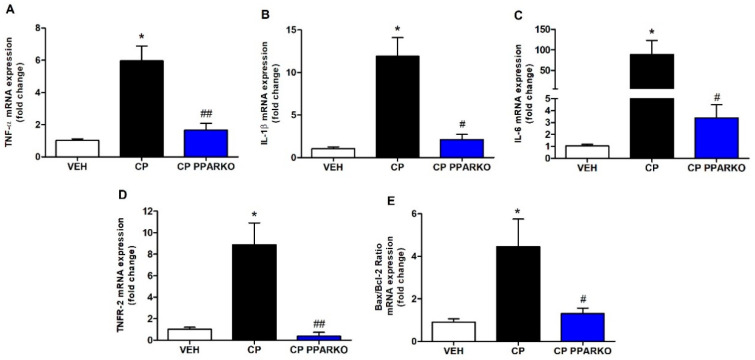
PPAR-α deletion attenuates cisplatin-induced increased pro-inflammatory cytokines and apoptosis-related genes. Cisplatin treatment (CP) increased mRNA levels of pro-inflammatory cytokines, (**A**) *TNF-α*, (**B**) *IL-1β* and (**C**) *IL-6* in renal tissue; PPAR-α knockout mice (CP PPARKO) prevented this increase. Apoptosis-related genes (**D**) *TNFR-2* and (**E**) *Bax/Bcl-2* ratio were also increased by cisplatin (CP) and PPAR-α deletion (CP PPARKO) avoided this increase. *n* = 5–6 per group. One-way ANOVA followed by post hoc Tukey’s test. * *p* < 0.05 compared to the VEH group. # *p* < 0.05; ## *p* < 0.01 compared to the CP group.

Several studies have evidenced that inflammation contributes to cisplatin-induced nephrotoxicity [1,2]. Proinflammatory cytokines are produced mainly by activated macrophages and are tightly involved in augmenting inflammatory reactions [21]. CP exponentially increased renal expression of *TNF-α*, *IL-1β* and *IL-6*, while CP PPARKO blunted these increases (Figure 1A–C). Cisplatin-induced renal cell death involves several pathways, including apoptosis. We performed qPCR for *TNFR-2*, which is related to apoptosis extrinsic pathway and *Bax/Bcl-2*, which is related to apoptosis intrinsic pathway. CP upregulated *TNFR-2* and *Bax/Bcl-2* in renal tissue and CP PPARKO avoided the upregulation of these apoptosis-related genes (Figure 1D–E).

### 2.3. PPAR-α Ablation Protects against Cisplatin-Induced Apoptosis and Tubular Injury

**Figure 2:**
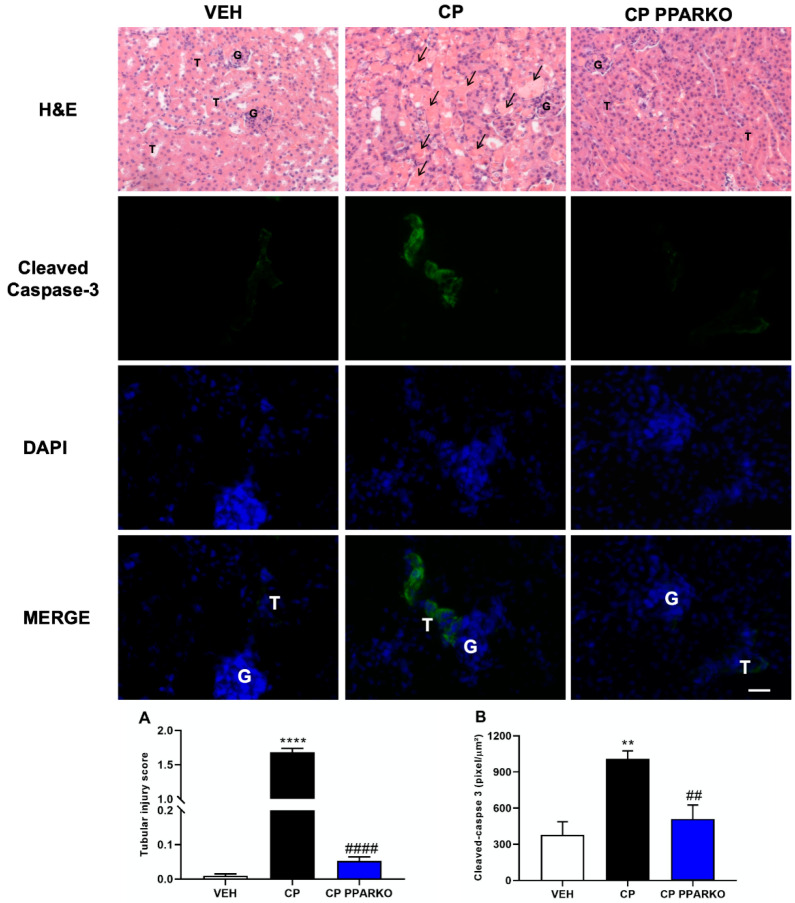
PPAR-α deletion attenuates tubular injury and apoptosis induced by cisplatin after 96 h. Representative photomicrography of H&E staining. (**A**) CP treatment increases tubular injury while PPAR-α deletion attenuates it. (**B**) Immunofluorescence was performed to assess apoptosis. CP increases cleaved caspase-3 staining and CP PPARKO reverses this increase. In arrows is indicated tubules with the tubular lumen obstructed by the tubular casts and cell detachment from the tubular basement membrane. G to indicate glomeruli and a T for examples of tubules with normal structure, no cell detachment and free tubular lumen. *n* = 5 per group. Scale bar = 100 µm. One-way ANOVA followed by post hoc Tukey’s test. ** *p* < 0.01, **** *p* < 0.0001. compared to the VEH group. ## *p* < 0.01, #### *p* < 0.0001; compared to the CP group.

Cisplatin affects the proximal tubules of the kidneys through several mechanisms, including tubular necrosis. In the histological analysis, we observed a large increase of tubular injury in cisplatin-treated mice, while PPAR-α knockout mice showed tubular cells protected against cisplatin-induced nephrotoxicity (Figure 2A–B). Cisplatin administration leads to increased apoptosis in the kidney. Caspase-3 is the main executioner caspase and is activated in the apoptotic cell, by both intrinsic and extrinsic pathways. Immunofluorescence was performed to identify apoptosis in renal tissue. Cisplatin treatment increased caspase-3 in wild-type mice and PPAR-α ablation prevented the increase of this protein (Figure 2B).

### 2.4. PPAR-α Deletion Prevents Downregulation of Organic Transporters Induced by Cisplatin

**Figure 3:**
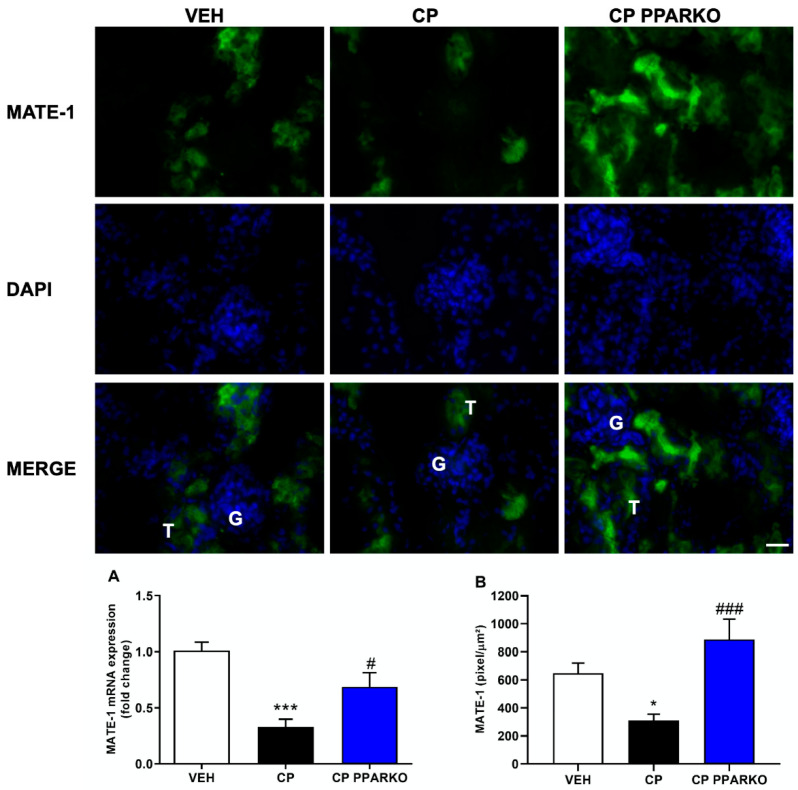
PPAR-α knockout mice mitigate the decreased mRNA and protein expression by immunofluorescence of MATE-1. Ninety-six hours after cisplatin treatment (CP) downregulates (**A**) mRNA and (**B**) protein levels of MATE-1. PPAR-α knockout mice (CP PPARKO) prevented this downregulation. G to indicate glomeruli and a T to indicate tubules. *n* = 5 per group. One-way ANOVA followed by post hoc Tukey´s test. Scale bar = 100 µm. * *p* < 0.05, *** *p* < 0.001 compared to the VEH group. # *p* < 0.05, ### *p* < 0.001 compared to the CP group.

**Figure 4:**
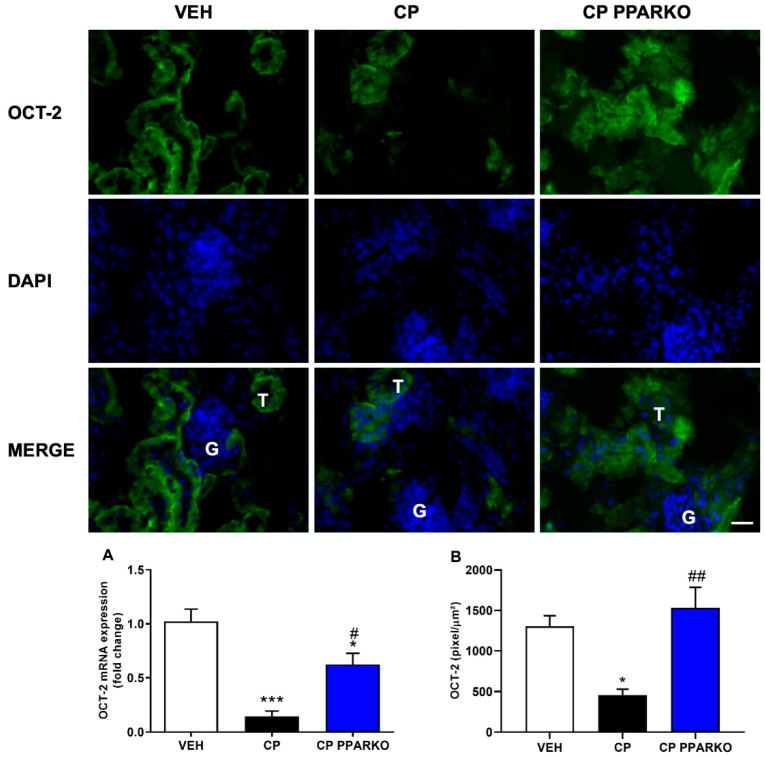
PPAR-α ablation attenuates downregulation of mRNA and protein expression by immunofluorescence of organic cations transporter 2 (OCT-2). Ninety-six hours after cisplatin treatment (CP) downregulates (**A**) mRNA and (**B**) protein (levels of OCT-2. PPAR-α knockout mice (CP PPARKO) attenuated this downregulation. G to indicate glomeruli and a T to indicate tubules. *n* = 5 per group. One-way ANOVA followed by post hoc Tukey´s test. Scale bar = 100 µm. * *p* < 0.05, *** *p* < 0.001 compared to the VEH group. # *p* < 0.05, ## *p* < 0.01; compared to the CP group.

Organic Transporters, such as OCT-2 and MATE-1, are of great importance in cisplatin nephrotoxicity: the first one is the main transporter of cisplatin into kidney cells, and the second one is responsible for cisplatin extrusion from the kidney into the urine. Cisplatin treatment induced the downregulation of OCT-2 and MATE-1 in the renal tissue, while PPAR-α deletion prevented cisplatin-induced downregulation of MATE-1 (Figure 3A–B) and OCT-2 (Figure 4A–B).

### 2.5. PPAR-α Knockout Modulates Organic Transporters

**Figure 5:**
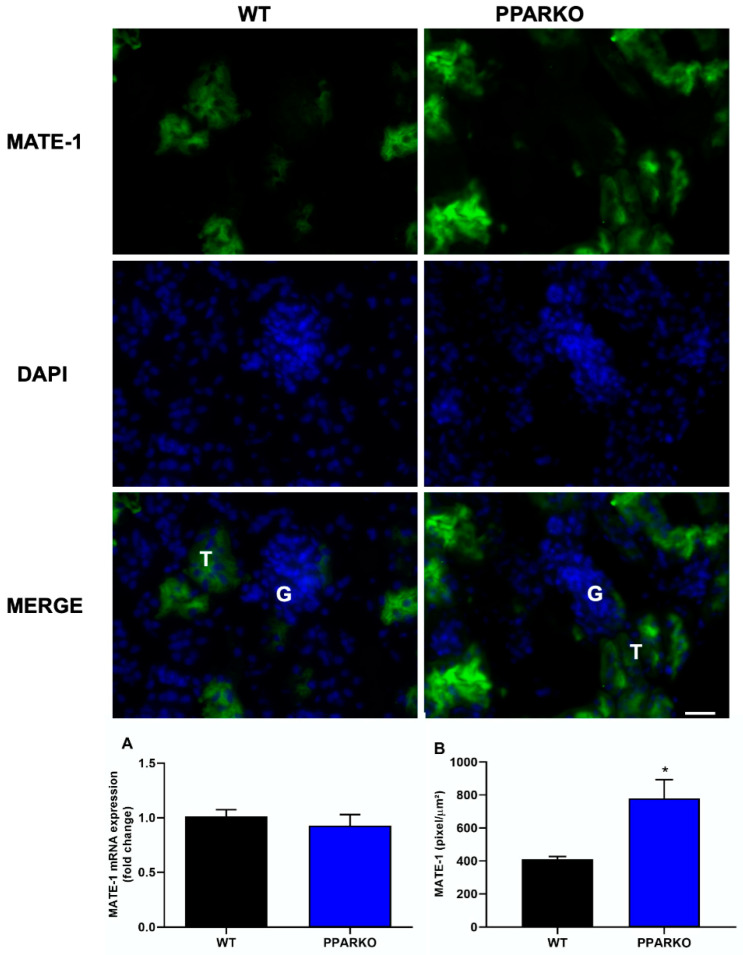
PPAR-α absence enhances protein expression by immunofluorescence of multidrug and toxin extrusion 1 (MATE-1). (**A**) No differences between WT and PPARKO mice were found in *MATE-1* mRNA levels. (**B**) However, PPAR-α knockout mice enhanced MATE-1 protein levels. G to indicate glomeruli and a T to indicate tubules. *n* = 5 per group. Scale bar = 100 µm. Two-tailed Student´s t-test. * *p* < 0.05, compared to the WT group.

**Figure 6:**
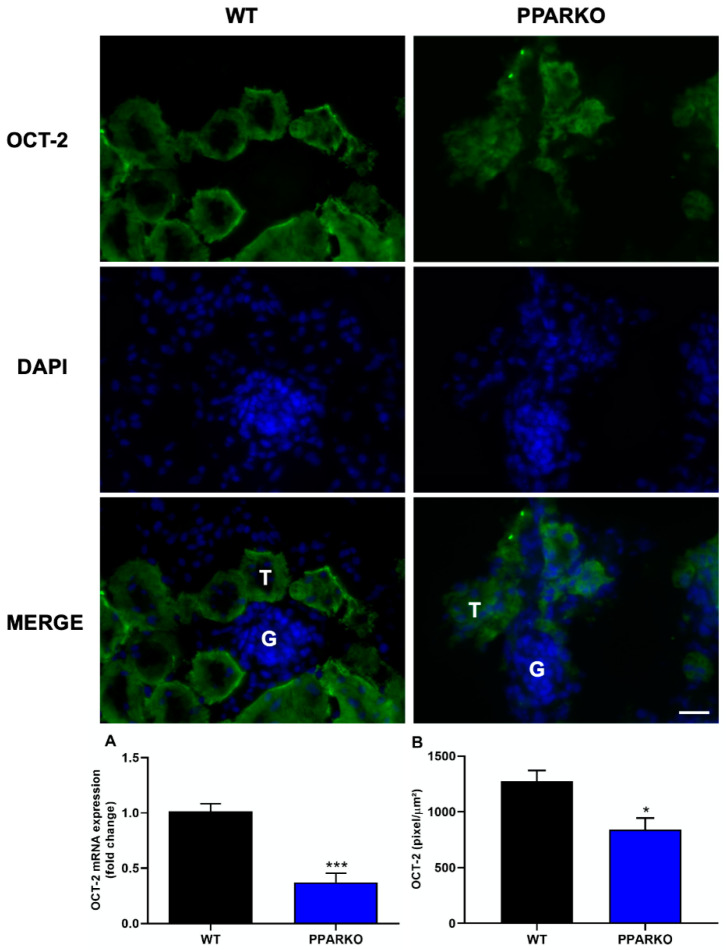
PPAR-α absence decreases mRNA and protein expression by immunofluorescence of organic cations transporter 2 (OCT-2). PPAR-α knockout mice presented reduced (**A**) mRNA and (**B**) protein levels of renal OCT-2. G to indicate glomeruli and a T to indicate tubules. *n* = 5 per group. Two-tailed Student´s t-test. Scale bar = 100 µm. * *p* < 0.05, *** *p* < 0.001 compared to the WT group.

Immunofluorescence and real-time PCR were performed to check organic transporters protein and mRNA levels at the basal state. PPAR-α absence did not alter MATE-1 mRNA expression (Figure 5A); however, PPARKO mice showed increased MATE-1 protein levels observed in immunofluorescence (Figure 5B). In addition, organic cations transporter 2 (OCT-2) mRNA expression and protein levels were decreased in PPAR-α knockout mice (Figure 6A,B).

## 3. Discussion

Cisplatin is one of the most potent chemotherapy drugs used against solid tumors. It has a high success rate after treatment, although nephrotoxicity affects about one-third of patients treated with it [22]. Membrane transporters, such as MATE-1 and OCT-2, are of great importance for mediating cellular transport of cisplatin [6]. Our group has recently shown that PPAR-α, a transcription factor highly expressed in the kidneys, which controls lipid metabolism and glucose homeostasis, interacts with both MATE-1 and OCT-2 [20]. The modulation of both membrane transporters should be better explored for the use of therapies that can reduce nephrotoxicity in cisplatin-treated patients.

Our data shows that PPAR-α deletion attenuates cisplatin-induced nephrotoxicity, mainly by modulating the expression of the membrane transporter responsible for cisplatin extrusion from the kidneys. We have previously shown that restoration of MATE-1 expression after cisplatin nephrotoxicity is very important to decrease platinum accumulation in renal tissue [17].

Here, we show that PPAR-α deficiency was capable of reversing renal dysfunction by decreasing serum creatinine and urea levels induced by cisplatin treatment. Kidney injury molecule-1 (KIM-1) is known to be a biomarker of renal proximal tubular injury and is markedly upregulated after acute kidney injury [23,24,25]. The production and release of neutrophil gelatinase-associated lipocalin (NGAL) from tubular cells after renal damage are increased and it has been a useful biomarker for assessing the severity of kidney injury [26,27]. In order to confirm that PPAR-α deletion protects against renal damage, we performed qPCR for *KIM-1* and *NGAL* and found that cisplatin treatment exponentially increased these markers in renal tissue, while PPAR-α knockout mice attenuated the increasement of this kidney damage markers after cisplatin exposure. In addition to direct cellular toxicity, inflammation plays an important role in cisplatin nephrotoxicity. Over the years, a number of mediators of inflammatory renal injury have been identified, and inflammatory cytokines have shown to be increased after cisplatin toxicity [28,29,30]. TNF-α plays an important role in many infectious and inflammatory diseases. TNF-α inhibition and deletion reduced cisplatin-induced renal injury and increased survival rates after its administration [28]. In our study, we observed that cisplatin treatment increased proinflammatory cytokines and that PPAR-α knockout mice can reverse it.

Multiple pathways and molecules are involved in cisplatin-induced nephrotoxicity and apoptosis is observed after cisplatin administration [31,32]. Apoptosis may occur in cisplatin treatment by activation of apoptotic pathways, such as intrinsic mitochondrial pathway and extrinsic pathway activated by death receptors [1,33,34]. TNFR-2 mediates apoptosis in cisplatin-induced injury and is one of the death receptors of the extrinsic pathway [35]. Moreover, the Bax/Bcl-2 ratio can be used to determine the intrinsic pathway of apoptosis [33,34]. Cisplatin treatment induced upregulation of *TNFR-*2 and *Bax/Bcl-2* ratio, while PPAR-α knockout mice are protected against this upregulation. We confirmed apoptosis by analysis of cleaved caspase-3, which is the executioner caspase. In addition to apoptosis, necrosis is commonly observed with cisplatin treatment. Indeed, our cisplatin treatment presented huge histological changes of acute tubular necrosis, while PPAR-α deletion was able to avoid it.

As PPAR-α ablation attenuates renal dysfunction, renal injury, inflammatory- and apoptosis-related markers, we further investigated if organic transporters may be involved in PPAR-α deficiency protection. We found that cisplatin induced downregulation of both MATE-1 and OCT-2 in renal tissue, while PPAR-α knockout mice restored the expression of both membrane transporters, important to note that this effect in WT mice may also be related with destruction and loss of tubules. MATE-1 is an important membrane transporter, responsible for cisplatin extrusion from the kidney into the urine [7,14], and its deletion exacerbates cisplatin nephrotoxicity [16]. Indeed, our data corroborates the study by Oda et al. who observed decreased OCT-2 and MATE-1 protein levels in renal tissue after cisplatin treatment. Decreased OCT-2 expression can delay platinum incorporation and diminished MATE-1 expression can increase platinum accumulation in renal cells; therefore, reduced expression of both transporters appears to enhance cisplatin accumulation in renal tissue [36]. We have previously shown that restored MATE-1 expression in renal tissue is important to decrease the renal toxicity induced by platinum accumulation [17]. In silico prediction of binding sites provides evidence that PPAR-α response elements (PPRE) regulate MATE-1 [37]. In addition, PPAR-α has been shown to regulate the transcription of OCT-2 gene: co-transfection of OCT-2 luciferase reporter construct with PPRE leads to a 10-fold increase in transcriptional activity [36]. Moreover, we found that PPAR-α knockout mice present reduced expression of OCT-2 and increased expression of MATE-1, which lead to less cisplatin available to enter renal cells and increase the capacity to extrude cisplatin from cells into urine.

Interestingly past works show that PPAR-α activation promotes protection in different models of kidney injury [33,38,39,40,41,42,43,44]. Indeed, its well stablish that increasement of lipid metabolism is beneficial in several models of diseases [45,46,47]. However, not much is discussed regarding other metabolic pathways compensation due to impaired lipid metabolism in PPAR-α deficiency. Further studies are required to better elucidated these mechanisms of metabolic compensation that drive PPAR-α deletion to promote protection against cisplatin nephrotoxicity.

Here, we show for the first time that PPAR-α deletion is capable of attenuating cisplatin-induced nephrotoxicity and that this is due to the restoration of both MATE-1 and OCT-2 expression, thus suggesting that increases cisplatin extrusion from the kidneys into the urine and decreases the direct toxicity caused by cisplatin accumulation in renal cells.

## 4. Materials and Methods

### 4.1. Animals

Littermates wild-type (WT, C57/BL6J) and PPAR-α knockout (PPARα KO, B6; 129S4-Pparatm1Gonz/J, Jackson laboratory) male mice weighing 23–27 g and aged 10–14 weeks were used for these experiments. The animals were obtained from the Animal Care Facility of the Federal University of São Paulo (UNIFESP). All animals were housed in individual, standard cages and had free access to water and food. All procedures were previously reviewed and approved by the internal ethics committee of the Federal University of São Paulo (CEUA 6823010319 issued on 5 June 2019).

### 4.2. Experimental Protocol

The mice were divided into 3 groups for each experiment: vehicle group (VEH), cisplatin (CP)-treated group and PPARα KO + cisplatin (CP PPARKO)-treated group. We used *n* = 5–6 for each experiment and condition, experiments were repeated 2 to 3 times.

### 4.3. Cisplatin Treatment

Single doses of cisplatin (20 mg/kg—Bergamo, Taboão da Serra, Brazil) were injected intraperitoneally. Tissues and blood were collected 96 h after injection. Vehicle group animals received 0.9% NaCl intraperitoneally at same volume as cisplatin.

### 4.4. Blood Sampling and Kidney Collection

The mice were anesthetized with ketamine (91 mg/kg) and xylazine (9.1 mg/kg) intraperitoneally and blood was collected via heart puncture. Blood was allowed to clot for 2 h at room temperature and then centrifuged for 20 min at 2000× *g*. The samples were stored at −20 °C. Kidney tissue was collected, and renal capsule was removed. Transversal cuts were performed, and the kidneys were stored at −80 °C.

### 4.5. Renal Function

Serum creatinine and urea levels were used to determine renal function. Samples were analyzed using commercially available colorimetric assay kits (Labtest, Lagoa Santa, Brazil).

### 4.6. RNA Extraction and RT-qPCR

Whole kidney total RNA was isolated using TRIzol Reagent (Invitrogen, Carlsbad, CA, USA). The RNA integrity was assessed by electrophoresis on an agarose gel. cDNA was synthesized using the “High Capacity cDNA Reverse Transcription Kit” (Applied Biosystems, Foster City, CA, USA). Standard curves were plotted to determine the amplification efficiency for each primer pair. Real-time PCR was performed using two systems: TaqMan system (Applied Biosystems, Carlsbad, CA) using probes for IL-6 (mm00446190-m1), YWHAZ (mm03950126-s1) and GAPDH (mm99999915-g1); and SYBR Green system (Thermo Scientific, Waltham, MA, USA) using specific primers for β-actin, 18s, IL-1β, NGAL, KIM-1, BAX, BCL-2, TNFR-2, TNF-α, OCT-2 and MATE-1; the primers were designed using primer3 web and their specificity was confirmed using NCBI primer-BLAST; their sequences are shown in Table 2. The cycling conditions for both TaqMan and SYBR Green reactions were as follows: 10 min. at 95 °C, followed by 45 cycles of 30 s at 95 °C, 30 s at 60 °C and 30 s at 72 °C. Target mRNA expression was normalized to both housekeeping genes, β-actin and 18s for SYBR and to YHWHAZ and GAPDH for TaqMan and expressed as a relative value using the comparative threshold cycle (Ct) method (2^−ΔΔCt^). The expression levels of the genes of interest were normalized to the vehicle group and presented as fold change.

### 4.7. Tubular Injury Analyses

The kidneys were fixed in 10% formaldehyde and then dehydrated and embedded in paraffin. Sections (4 µm) were cut and stained with hematoxylin–eosin. At least six subcortical fields were visualized and analyzed for each mouse using a (Zeiss, Oberkochen, Germany) microscope at a 200× magnification. Tubular injury score was determined based on the percentage of tubules showing luminal casts, cell detachment or dilation and assigned according to the following scale: 0 = 0 to 5%, 1 = 6 to 25%, 2 = 26 to 50%, 3 = 51 to 75% and 4 > 75%.

### 4.8. Kidney Extraction and Sectioning

The kidney was harvested and then cryoprotected for additional 2 days by immersion in 30% sucrose at −20 °C. Acetone-fixed cryosections (7 μm; Cryostat-Leica Biosystem, Wetzlar, Germany)) were mounted for immunofluorescence analysis.

### 4.9. Immunofluorescence

The immunofluorescence was performed according to Cavalcante et al. 2019 [48]. Briefly, after fixed with −20 °C acetone the kidney sections were incubated with primary mouse anti-cleaved caspase-3 antibody (1:300, Cell Signaling, Danvers, MA, USA #9661S), anti-MATE-1 antibody (1:200, Santa Cruz, Dallas, TX, USA, sc-138983) or anti-OCT-2 antibody (1:250, Boster Bio, Pleasanton, CA, USA, PB9394) overnight at 4 °C. Nonspecific binding was controlled by replacing a negative control with the primary antibody. After this, the sections were incubated with Alexa Fluor 488 anti-rabbit (1:300, Thermo Fisher, #A11034) during 2 h. The nuclei were stained with DAPI (1:2000, Thermo Fisher, #D1306). Finally, the slices were coverslipped in Mowiol (Sigma-Aldrich, San Luis, MO, USA) mounting media. Sections were imaged in Zeiss fluorescence microscope (Zeiss, Oberkochen, Germany) using a 488 nm excitation. During the microscopic analysis, an overview was performed to qualify the slides, then 10 images were acquired employing a 10× objective, and finally, a representative image was acquired using a 20× objective. The fluorescence intensity was analyzed using ImageProPlus software (version 4.0) and the results were presented as fluorescence intensity/area. Pictures were taking using exactly the same illumination conditions. It is worth to note that all these procedures were performed in a double-blind manner.

### 4.10. Statistical Analysis

All data are presented as mean ± SEM. Intergroup differences significance was assessed by one-way analysis of variance (ANOVA) with the Tukey’s correction for multiple comparisons. The value for statistical significance was established at *p* < 0.05. All statistical analyses were performed using GraphPad Prism 8 (GraphPad, La Jolla, CA, USA).

## Figures and Tables

**Table 1:** Effects of peroxisome proliferator-activated receptor alpha (PPAR-α) deletion on renal injury and function.

Title	VEH	CP	CP PPARKO
Parameters	Mean ± SEM	Mean ± SEM	Mean ± SEM
Creatinine (mg/dL)	0.591 ± 0.023	3.639 ± 0.611 ***	0.853 ± 0.063 ###
Urea (mg/dL)	62.62 ± 1.512	575.6 ± 48.39 ***	109.4 ± 17.50 ###
NGAL mRNA expression	1.105 ± 0.235	308.1 ± 55.39 ***	71.04 ± 24.56 ##
KIM-1 mRNA expression	1.160 ± 0.311	732.8 ± 88.50 **	163.2 ± 54.69 #

Data are presented as mean ± SEM. ** *p* < 0.01, *** *p* < 0.001. compared to the VEH group. # *p* < 0.05; ## *p* < 0.01; ### *p* <0.001. compared to the CP group.

**Table 2:** Sequences of the primers used for real-time PCR assays.

Primers for RT–PCR	xxx	xxx
*Gene*	Forward 5′-3′	Reverse 5′-3′
*18S*	CGC CGC TAG AGG TGA AAT TC	TCT TGG CAA ATG CTT TCG C
*β-actin*	CTG GCC TCA CTG TCC ACC TT	CGG ACT CAT CGT ACT CCT GCT T
*BAX*	CGG CGA ATT GGA GAT GAA CTG	GCA AAG TAG AAG AGG GCA ACC
*BCL-2*	ACC GTC GTG ACT TCG CAG AG	GGT GTG CAG ATG CCG GTT CA
*IL-1β*	AGG AGA ACC AAG CAA CGA CA	CGT TTT TCC ATC TTC TTC TTT G
*KIM-1*	TGT CGA GTG GAG ATT CCT GGA TGG T	GGT CTT CCT GTA GCT GTG GGC C
*MATE-1*	AGG CCA AGA AGT CCT CAG CTA TT	ACG CAG AAG GTC ACA GCA AA
*NGAL*	ATG TGC AAG TGG CCA CCA CG	CGC ATC CCA GTC AGC CAC AC
*OCT-2*	AGC CTG CCT AGC TTC GGT TT	TGC CCA TTC TAC CCA AGC A
*TNF-* *α*	GCC TCT TCT CAT TCC TGC TTG	CTG ATG AGA GGG AGG CCA TT
*TNFR-2*	GTC GCG CTG GTC TTC GAA CTG	GGT ATA CAT GCT TGC CTC ACA GTC

## Abbreviations

CP	Cisplatin
GAPDH	Glyceraldehyde 3-phosphate dehydrogenase
IL-1β	Interleukin-1 beta
IL-6	Interleukin-6
KIM-1	Kidney injury molecule-1
MATE-1	Multidrug and toxin extrusion protein 1
NGAL	Neutrophil gelatinase-associated lipocalin
OCT-2	Organic cation transporter-2
PPAR-α	Peroxisome-proliferator-activated receptor alpha
PPARKO	Peroxisome-proliferator-activated receptor alpha knockout
PPRE	Peroxisome-proliferator-activated receptor alpha response elements
TNF-α	Tumor necrosis factor alpha
TNFR-2	Tumor necrosis factor alpha receptor-2
VEH	Vehicle control group
BAX	B-cell lymphoma 2 associated x protein
BCL-2	B-cell lymphoma 2
WT	Wild-type
YWHAZ	14-3-3 protein zeta/delta
